# “Palliative care is so much more than that”: a qualitative study exploring experiences of hospice staff and bereaved carers during the COVID-19 pandemic

**DOI:** 10.3389/fpubh.2023.1139313

**Published:** 2023-10-25

**Authors:** Cara Bailey, Ping Guo, John MacArtney, Anne Finucane, Richard Meade, Susan Swan, Ellie Wagstaff

**Affiliations:** ^1^School of Nursing and Midwifery, University of Birmingham, Birmingham, United Kingdom; ^2^Unit of Primary Care, University of Warwick, Coventry, United Kingdom; ^3^Clinical Psychology, University of Edinburgh, Edinburgh, United Kingdom; ^4^Marie Curie Hospice, Edinburgh, United Kingdom; ^5^Clinical Psychology, Carers UK, Edinburgh, United Kingdom; ^6^Maggie's Glasgow, Glasgow, United Kingdom

**Keywords:** palliative, COVID, hospice, public health, workforce, end-of-life, policy change, qualitative

## Abstract

**Background:**

The way in which end-of-life care was provided changed significantly during the first 2 years of the COVID-19 pandemic. The national lockdown restrictions reduced formal care support services and increased the burden on many carers taking on the caring role for the first time. We aimed to explore decision-making about the place of care during the COVID-19 pandemic and the impact on experience from the perspectives of carers and hospice staff caring for people at the end-of-life.

**Methods:**

A qualitative study using virtual interviews was conducted between October 2020 and April 2021. Data were analyzed thematically using framework analysis, an analytical framework that enables qualitative research to be organized into defined themes derived from the research question. Findings were presented to stakeholders in policy roundtables between March 2022 and March 2023 and discussed collaboratively with staff, stakeholders, and the public to inform policy and practice change.

**Findings:**

A total of 37 participants (15 bereaved carers and 22 staff) were recruited via hospice services in England and Scotland. Four key themes were identified: (1) changing preferences relating to decision-making about the place of care and the impact at the time of death and into bereavement; (2) missed opportunities related to not being there, not having others around, and being robbed of memory-making; (3) the lone carer during a period of high intensity and reduced home support; (4) process vs. person-centered care resulting from changing rules and restrictions and prioritization of regulations over essential palliative care.

**Conclusion:**

The study provides valuable global implications for all involved in end-of-life care. Despite great efforts to provide dignified, quality care, palliative care during the pandemic changed, focusing on essential ‘physical care'. The psychological suffering experienced by staff and carers may need longer-term support mechanisms put in place, which will benefit from a public health approach. Policymakers should consider improving carer identification and resources for wider end-of-life care education to support the needs of carers, health and social care staff, and citizens.

## Introduction

As the population ages, we are seeing more people live to an older age with complex health problems (1), from which they are likely to die. Until recently, non-communicable diseases accounted for over two-thirds of all global deaths (2). However, in a global pandemic, the projections on which decisions about resource allocation and service delivery are based may not be accurate. Projections for the first 10 weeks of the pandemic predicted an increase of 220% in deaths in care homes, a 77% increase at home, and 90% in hospitals, but hospice deaths fell by 20%. Bone et al. (3) found that excess deaths were amongst older people (86% aged ≥ 75 years) and estimated that 22% (13%−31%) of COVID-19 deaths occurred amongst those at the end-of-life. Identifying where people die and understanding the decision-making process behind end-of-life care is important in order to support health policies, resource allocation, and commission services. The “Better end-of-life programme” in the UK (4) highlighted how major shifts in place of end-of-life care along with population aging will place substantial demands on palliative care services over the next 20 years.

During the COVID-19 pandemic, face-to-face health and social care provision was restricted, intensifying the burden on carers. UK statistics suggest that during the pandemic, there were over 13.6 million such people providing care through the pandemic (5). Even before the pandemic, ill health was reported amongst carers, but recent figures suggest further negative impacts on carers with 67% of carers on NHS waiting lists for poor mental and physical health (5). The impact of a pandemic where visiting was restricted and social gatherings stopped during national lockdown is likely to have increased the number of people taking on caring roles and exacerbated the impact on health and wellbeing. The report ‘Caring behind closed doors' (5) estimates that 70% of carers provided more care during the pandemic (on average, an additional 10 h per week). There were continual concerns about who would care for their loved one or person they cared for if they got ill or had to self-isolate, with 55% of carers feeling overwhelmed and worried about burnout due to caring responsibilities (5). For those caring for someone with a terminal illness during the first 18 months of the pandemic, the move to communities away from hospitals and inpatient hospice care (6) meant that family members, companions, and friends became carers taking on end-of-life care responsibilities in their own homes.

Globally, COVID-19 had a profound impact on health and social care professionals. Pre-pandemic, hospices experienced significant challenges with increased patient acuity, staffing, and funding challenges (7). The evidence that is available to date post-pandemic shows that these challenges are amplified, and hospice services were forced to reconfigure to meet the demand for specialist palliative care (8). Whilst hospice inpatient admissions were reduced, community care significantly increased, and hospice staff responded to the change through shifting resources, upskilling care staff, and remote working (9). Reports of staff shortages, concerns over shortages of personal protective equipment (PPE), and managing increased staff anxiety around the pandemic have put immense pressure on already overwhelmed hospice services in the UK (4), Italy (10), and the USA (11, 12). Hospice out-of-hours services were also challenged due to a lack of integration within wider healthcare services and workforce issues (9). What remains unknown is the impact of the changes in the quality of care from the perspective of those providing the care, which this study aimed to address. The study was necessary to explore the experiences of the hospice paid and unpaid workforce during the national lockdown period in the UK, which had not previously been investigated or reported. The qualitative approach enabled a rich and deep understanding of decision-making and the impact of such decisions on practice and life experiences. The study is important to provide an evidence base to inform policy and practice change in order to better support people at the end-of-life and those that care for them. Despite being conducted in the UK, the article reports wider implications for all involved in end-of-life care (and for an international context) given the insights into how care was “experienced”, the decisions that were made about the place of care and the impact it had on bereavement and beyond.

### Aim

We examined the decision-making behind the place of end-of-life care during the COVID-19 pandemic and the impact it had on the experiences of end-of-life care from the perspectives of carers and hospice staff. We had three aims to explore:

(1) the decision-making about the place of care during the pandemic,
(2) the impact on the quality of hospice care, and
(3) the end-of-life experiences of hospice staff and carers.

This article reports the key findings in relation to the aims, highlighting recommendations for public health, policy, and practice. Standards for Reporting Qualitative Research (SRQR) guidelines have been used to ensure sufficient detail in reporting (13).

## Materials and methods

### Design

A qualitative study was conducted to enable a rich, in-depth understanding of the views and experiences of the hospice staff and unpaid carers during the COVID-19 pandemic. The research was underpinned by the philosophical construct of critical realism (14), a particularly useful stance to understand how and why things happen and unpack the influence of context on the outcomes in a natural setting. In this research, we were particularly interested in people's decisions during a period of crisis (both end-of-life and a global pandemic) and their behaviors toward changing processes involved in their care and support systems, which the critical realistic approach enabled.

### Patient and public involvement

Three members of the BRHUmB Palliative Care Research Hub Experts by Experience were involved in the study (see: https://www.birmingham.ac.uk/schools/nursing/research/brhumb/index.aspx). All had previous experience providing care to someone at the end-of-life. PPI members advised on the study design and recruitment, participated in mock interviews, and fed back on preliminary findings prior to the final themes being confirmed and presented in the roundtable discussions. Two members (MN and AF) have been involved in the preparation of the article and will continue to be part of its dissemination; the other member unfortunately died during the study period. PPI members were paid for their involvement based on NIHR guidance and were supported by CB.

### Population and sampling procedure

#### Staff

We planned to recruit 20 hospice staff. Staff were purposefully recruited from hospices and community care services in England via research nurse (RP) and in Scotland via clinical nurse specialist/researcher (SS). A sampling frame was developed in an attempt to capture a broad range of roles at each site (nurses, doctors, social workers, pharmacists, leads of services, e.g., education, bereavement leads, and managers) and experiences (e.g., years in role, community and inpatient settings, and night and day services). Recruitment was supported by research facilitators JM in England and AF in Scotland to ensure staff were not overburdened with ongoing research activity. Eligible staff had to be working in hospice care (community or inpatient hospice unit), covering part of the second lockdown period of October 2020–April 2021. RP and SS approached staff on-site to provide information about the study, answer questions, and give staff the opportunity to participate in the study. Staff were given an information sheet and permission for their details to be handed to CB (in England) to arrange an interview or an interview arranged directly with SS (in Scotland).

#### Bereaved carers

We planned to recruit 25–30 bereaved carers. Eligible carers had to have cared for someone who died at home at least three months prior to recruitment, and death had to have occurred during the period October 2020–April 2021. We felt the time of the second national lockdown in the UK would capture the most extreme impact due to the restrictions on both formal and informal support services and networks.

At the English site, bereaved carers were purposefully recruited via the hospice bereavement service, with the bereavement lead ensuring eligibility and providing contact details. In Scotland, the community nurse specialists at each site were asked to identify and contact eligible carers who may be interested in participating, as was the hospice bereavement service.

In Scotland, the hospice telephone bereavement service was also asked to contact eligible carers. Once permission had been given, a follow-up telephone call was conducted by SS, and information was emailed or posted. Newspaper articles in local and national articles and social media posts were also used to raise awareness and encourage interested carers to contact SS. At each site, RP and SS contacted the carers in a follow-up telephone call, which is often conducted by the bereavement team as a way of offering further bereavement support if any is required. During the call, RP and SS informed the carers of the study and asked whether they would consider being part of it. If they wished to find out more, an information sheet was emailed or a paper copy was posted to them. Participants could then directly contact the lead researcher CB in England or reply to SS in Scotland if they wished to participate in an interview, or alternatively contact the research nurse (RP) for more information. Participants at both sites were excluded if they were not able or willing to provide informed consent. Any carers who did not wish to take part but appeared to need further bereavement support were directed to bereavement support groups or their GP. All participants taking part were offered a £20 voucher for an online shopping store as a gesture of thanks for their participation.

### Data collection

Consent was provided online prior to data collection. Participants were asked to email a copy of the signed consent form to the researcher prior to the interview if they were able to do so. For those unable to email in advance, a link was provided to an online consent form that was signed prior to the interview starting. Consent was re-confirmed verbally by the researcher at the start of the interview, and the online form was checked and confirmed. Interviews were conducted online (via Zoom or Microsoft Teams) or over the telephone (depending on the preference of the participant). Most participants conducted the online interview from their own homes; some staff used a private office space within the hospice. Interviews (lasting between 45 and 90 min) were conducted by SS, CB, and PG using the record function in zoom/teams or via dictaphone for telephone interviews. A semi-structured interview schedule explored decision-making about care options and place of death, systems of support, and a reflection on the experience (Supplementary Files 1, 2).

Staff interview schedules explored the following: (1) impact on hospice care services; (2) impact on role and patient care; (3) decision-making about place of care; (4) visiting restrictions; (5) impact on bereavement and support for carers; and (5) quality improvement.

Carer interview schedules explored the following: (1) experience of hospice services; (2) impact of the pandemic on care; (3) influence on decision-making about the place of care and support, including access to other services, e.g., GP, visiting restrictions, out-of-hours support; (4) support; and (5) any restrictions or changes to care.

We also asked carers to complete a quality of life outcome measurement scale specifically designed for close people to the dying (ICECAP-CPM See: https://www.bristol.ac.uk/population-health-sciences/projects/icecap/icecap-cpm/) to allow further exploration of key aspects considered important to carers at the end-of-life (15). The scale includes questions about communication, privacy and space, emotional and practical support, preparation and coping, and emotional distress, designed to capture the benefits of end-of-life care for close people. These are considered to be the most important attributes for someone close to dying (15), and responses can be used to value the quality of care received (analysis reported elsewhere). Each question was presented on screen with five possible responses demonstrating capability ranging from fully able to completely unable. Slides were emailed to those who took part on the telephone. For each question, participants were asked to “think aloud” their answers, reflecting on their experiences. The “think-aloud” style enabled people to open up about their experiences and reflect on the impact (16).

Personally identifiable information was removed from the transcripts, which were psudononymised using a number system, e.g., for bereaved carers England (BCE_) and Scotland (BCS_) and staff in England (SE_) and Scotland (SS_), to enable analysis across and between data sets. Participants who knew the interviewer were therefore offered the opportunity to be interviewed by another researcher in the team, but no one took that option. Interview data were recorded (on the Zoom or Teams transcript or via dictaphone for telephone interviews). Transcripts were downloaded and converted to Word with errors cleaned and terms clarified (from the audio recording) to enable coding and analysis.

### Data analysis

Interview transcriptions were analyzed thematically using framework analysis (17). The framework method uses a systematic approach to categorize and organize large data sets and is particularly useful where multiple researchers are working on a project, as in this case. Data were initially analyzed by CB, an experienced qualitative researcher, and SR (under CB's supervision). After the first six transcripts, PG, CB, and SR organized and agreed on the coding matrix (spreadsheet) along with extracts from the transcripts under each of the codes (e.g., see Table 1 for an example of a small section from the decision-making theme and codes to illustrate matrix building).

**Table 1:** Example from coding matrix.

**Theme**	**Sub-theme**	**Code**	**Participant**	**Quote extract**
Changing preferences	Place of care	Avoid hospices, care homes, or hospitals due to fear of contracting virus	SS1	So I would think that people would be slightly anxious about the thought of coming into Hospice care during a pandemic, for all of you know the obvious reasons for getting COVID-19…..
			SS4	I would say right from the beginning it was evident that people were frightened to come to [local hospital]. They do not want to go into hospital and we used to say, look that is fine, we agree. I would not want to go in either as it was rife in hospitals and all the red wards and no visiting had a massive impact on people...That was a bigger issue for them. They did not want to leave the house, and when they were dying, the fact that they could not visit when they were in any hospital had a major impact on decisions as well.
			SE10	Huge reluctance to be admitted to a 24 h care facility, e.g., nursing home
		Visiting Restrictions	SE9	There were restrictions on visitations
			SE8	No visitation in the IPU
			SE10	Because of the visiting, the way people with COVID-19 were placed in there, and the way the media portrayed the nursing homes
		Stressful decision-making for staff about the number of visitors	SS1	Thinking about requests where you might have multiple family members wanting to visit and say goodbye.. the utter stress of that for staff... those horrible decisions. No one wants to be making those decisions and gatekeeping, an added level of stress that we did not have before the pandemic
			SS3	So I remember distinctively I am going to see a lady whose son was in [other non-MC hospice] dying. And whose husband needed to come in to here and was dying, and she could not go and see either. I am asking families to make these decisions just horrendous. So that was a…..that was probably one of the hardest things to personally cope with from the nurse perspective about people dying alone, not seeing their families, so we were desperately trying to keep people at home and not bring them in here.

Data were added to the matrix and further refined by CB and SR. PG reviewed the coding in the matrix and, with CB, developed themes by interrogating data categories through comparison between and within the cases to ensure accuracy and consistency. In several instances, a number of categories were merged into one theme until the key themes (with the most codes and sub-themes) were evident (See Table 2).

**Table 2:** Themes and sub-themes.

**Theme**	**Sub-theme**
Changing preferences	Place of Care—They did not want to come in
	Impact—on everything
Missed opportunities	Not being there
	Not having others around
	Restrictions on making memories
The lone carer	High Intensity
	Reduced support at home
	Increased quality time
Procedure vs. patient-centered care	Changing rules and regulations
	Essential care

### Cross-validation and policy implications

Preliminary findings were presented to the wider research team, and Marie Curie policy leads to reflect on, explore, and confirm the final key themes. Once data analysis was validated within the wider team, findings were presented in roundtable discussions organized by EW with stakeholders, policymakers, hospice providers, and service leads who manage local commissioning relationships to enable the findings to be discussed, influence plans for service delivery, and draw out broad policy implications from the analysis for hospice providers. Findings were compared to previous evidence to identify any changes in assumptions and behaviors since the pandemic. This phase is important to unpack the context and decision-making process for hospice use by listening to individual stories and rationales rather than relying on data sets alone that report the place of death reported in other studies.

### Ethical considerations

Ethics approval was obtained from Science, Technology, Engineering and Mathematics Ethical Review Committee, University of Birmingham: ERN_21-0205. Ethical aspects of implementation were considered within the research team, and research governance approval was obtained from each hospice site. A distress protocol based on the work of Draucker (18) was designed by CB and SS (Supplementary File 3) to refer to during the interviews if participants experienced emotional distress and to ensure participants had follow-up support where required when the interview ended. Participant anonymity has been protected by referring to “staff” rather than specific role given the small number of some professional roles at hospices, i.e., pharmacist, social worker, bereavement lead. A breakdown of participants by ID, role and from each site has not been provided either for the same reason.

## Findings

The final sample included 22 hospice staff across community and inpatient settings, including staff nurses, clinical nurse specialists, specialty doctors, consultants, pharmacists, occupational therapists, social workers, healthcare assistants, and leads in bereavement and education. There was a range of hospice experience from 1 year to over 30. A breakdown of staff roles is not provided to protect the anonymity of staff at each site, given the small number of specialist roles. No staff approached by researchers (SS and RP) refused participation.

Bereaved carers (*n*−15) included sons and daughters, wives, husbands, and partners. There was a range in ages from 32 to over 78. The majority identified as white British, which is comparable to the main population accessing hospice services in the UK. Only two bereaved carers from all that were approached refused participation, giving the reason of timing not being appropriate. Both were given details for follow-up bereavement support from the hospice. The Scottish sample had representation from both rural and urban areas across Scotland. The majority of carers were in the caring role for the first time.

Four key themes were identified from the staff and carer data:

(1) Changing preferences relating to decision-making about the place of care and the impact that had at the time of death and into bereavement;
(2) Missed opportunities related to not being there, not having others around, and being robbed of memory-making;
(3) The lone carer during a period of high intensity, reduced home support, which whilst challenging enabled quality time;
(4) Procedure vs. person-centered care resulting from changing rules and restrictions and prioritization of regulations over holistic palliative care.

Each theme is discussed using extracted data from the interview transcripts.

### Changing preferences

#### Place of care—“They didn't want to come in”

The COVID-19 pandemic had a major influence on preferences for place of care at the end-of-life mostly based on the opportunities for visiting. Even where inpatient hospice care had been perceived as the most ideal place of care, the inability to visit due to government guidance meant that more people were cared for at home, with the ultimate responsibility falling to family carers.

“*Because of the visiting restrictions, they didn't want to come in, they had complex palliative care needs but they refused to come in, not just the patients but relatives too saying if you go in I won't be able to see you. Visiting had a huge impact on people coming in and still is. At one time here [at the hospice] we would have people, 10 in a room, having a party, or someone with them 24/7 and we couldn't have that” (Staff 13)*.

Family members worried they would not see the person they were caring for again as visitors were not allowed into the hospice for large periods of time and rules about visiting changed frequently. Fears of being alone at the end-of-life, or experiences of being isolated during previous hospital or hospice inpatient admissions during the pandemic, affected people's decisions to remain at home to die.

“*He had nobody, he was on his own in a room (in hospital), it was total isolation. To the point that at the end when they said do you want to come in to the hospice or stay at home, I said can we visit? And they said no and I said no then, no way” (Carer 2)*.

“*They (hospice very quickly shut it (visiting) down so children couldn't go in. We couldn't get (young son) in to see his mum” (Carer 13)*.

Consequently, many tried to care for their relatives at home, taking on the role of family carers, which they may not have previously expected or been prepared for.

“*She took a lot of persuading to be cared for at home because she thought it would be hard for us, but she couldn't have us all visit” (Carer 1)*.

“*It was incredibly tough, juggling end-of-life care, work, covid, at the time a 6 year old, all those things was just a hell of a lot. We had people coming in but I found it very difficult” (Carer 13)*.

Even when carers knew the quality of care they would be able to give at home was less than what their family member may have received in hospices or where dignity may have suffered, they tried to support keeping them at home, some felt angry at not having a choice.

“*I had to help him with personal care things which he did not want me to have to do, if he'd been in the hospice someone else would have done that” (Carer 10)*.

“*People obviously did not want to come in and not be able to see their families. So they were choosing to stay at home even if that meant probably most of their medical care wasn't going to be as good as if they'd come in [to the inpatient unit]” (Staff 20)*.

During the lockdowns, people chose to stay at home, hospice inpatient admissions were reduced, and social interaction was limited, which left many carers and staff feeling that inpatients had become isolated.

“*With covid, we can't bring patients together. We don't have a communal area and that's therapeutic. We weren't able to socialize patients it increased their isolation along with the fact they weren't getting visitors in the same way” (Staff 5)*.

“*Dad died at home… initially he wanted to die in the hospice, somewhere peaceful not at home… but he never had his home comforts, his kindle, his netflix and spent a lot of time lonely in his room… you weren't allowed to visit and it reinforced his decision [to go home]” (Carer 5)*.

#### Impact “on everything”

All staff felt that although the impact of the pandemic on hospice care and hospice staff was hugely significant, this has largely gone unnoticed by society.

“*It has impacted massively in many ways that we haven't quite come to grips with” (Staff 23)*.

Staff having symptoms or being in contact with someone with symptoms led to staff shortages, which in turn meant a reduction in inpatient admissions to the hospice because there were not enough staff to care for patients on the unit.

“*We might not have enough beds available because we've got staff off and part of that is covid and people being in contact and having to isolate so it means reduced capacity and it lessens the likelihood of patients being able to come in” (Staff 1)*.

Staff felt patients were not getting admitted in a timely manner to the hospice; many were on admissions lists for days and had died before a bed was available, mostly due to reduced staff and resource capacity.

“*Patients haven't been able to come into the hospice who usually would have, patients on waiting lists who have died before they were meant to come over to us” (Staff 22)*.

“*There was no space at the hospice for her” (Carer 13)*.

Despite striving to offer choice to people at the end-of-life in terms of preferences for place of care, staff observed a drastic change in where people died and that “choice” was not an option.

“*The [reduced] beds were full, people weren't moving, and so that choice of where would you like to die changed and there actually wasn't a choice. So we were having to prepare patients and families, whether they liked it or not to stay at home and that was hard for lots of reasons” (Staff 3)*.

The reduction of inpatient admissions particularly changed the “culture” of the hospice setting that staff noticed. Many also commented that the patients who were admitted had more complex issues.

“*Hospice admissions seem more unwell than pre-pandemic admissions, more at a crisis point” (Staff 22)*.

Especially in later waves, where patients had put off accessing service-led support or had been unable to and symptoms had exacerbated.

“*I think she would have benefitted going to the hospice four days earlier” (Carer 13)*.

With news of the virus spreading, lockdown measures increased (although at different rates across the UK), and preferences changed rapidly, as did practice. Staff reported a noticeable difference in the way they were working, particularly in places where telephone services took over face-to-face visits. In areas of Scotland, home visits resumed earlier than in England, where telephone and video calls were the initial platform of assessment.

“*We were only visiting (the home) if it was absolutely unavoidable. We were assessing symptoms over the phone and that for us, was a massive change” (Staff 18)*.

For staff, making decisions about visitors and advising people about care became stressful, with some resulting in symptoms of burnout and having to take time off work.

“*Thinking about requests where you might have multiple family members wanting to visit and say goodbye... the utter stress of that for staff... those horrible decisions. No one wants to be making those decisions and gate keeping, it's an added level of stress that we didn't have before the pandemic” (Staff 13)*.

“*I had eight weeks sick leave due to burnout…psychological support for staff is needed beyond the ones in place for families and carers” (Staff 22)*.

It is clear from what bereaved carers and hospice staff have told us that the pandemic has had a major influence on where people at the end-of-life decided to be cared for and that was driven by visiting restrictions. Evidence here suggests that fewer inpatient beds were available due to staff shortages (as staff shielding, isolating, or redeployed), reduced funding for inpatient beds, and the restrictions that encouraged sequestering vulnerable people at home. Consequently, deaths moved into the community setting (in people's homes and care homes), but the structural and systemic issues meant that people felt their choice was removed.

The next stage of data analysis explores the impact in more detail and identifies three further themes of missed opportunities: the lone carer and procedure vs. patient-centered care.

### Missed opportunities

#### Not being there

Visiting restrictions led to carers feeling like they missed opportunities to be together. Not being there at the time of death or at important moments, such as communicating a terminal diagnosis or deterioration, was difficult for the carers. Carers described how they struggled to come to terms with not being with their family member at the end of their life. Particularly those who were admitted into the inpatient hospice or hospital from home felt traumatized by the experience of not being able to go with them.

“*I still felt utterly traumatized by the whole experience [crying], I just felt nobody cared [in hospital]. It ought have been better… I don't think there was any good reason to keep relatives of terminally ill people out of hospital. The staff were coming and going anyway, so what difference would it have made in terms of the covid situation? I can't see how it would have. It could have been better, even the staff didn't like what they were seeing” (Carer 14)*.

“*I will be critical of the care [in hospital], it was not good. There were too many patients to staff ratio. She'd be in there [hospital] for days, a week at a time and she'd say I've not seen anyone for hours, no one has come round, my pain medications is late. We couldn't go in to hospital so we were phoning saying get someone to give her pain meds now, that was on the general wards and that was not good. It was incredibly difficult. One time, I couldn't go in, I couldn't speak to her. The doctors and nurses are hardworking and really busy, I don't want to disturb them but they weren't taking care of her. I had to go through a complaints process to speak to someone to understand what was going on. Once I got through it completely changed but it was the absence of information of what was going on, that was awful. That communication was really poor” (Carer 13)*.

Not being able to go into clinical settings and advocate for loved ones or contact staff caused a great deal of distress and frustration for carers. One carer reflected on when her husband was admitted to the hospital and she could not contact them with important blood results from the GP, which warranted a change in his treatment.

“*I had the results that they (the hospital) needed but I couldn't' get through to the ward on the phone. I went to the hospital and they wouldn't let me in” (Carer 14)*.

Views of the hospice were more positive than those of the hospital but not being able to freely visit or stay with those at the end-of-life was frustrating for staff enforcing visiting restrictions in the hospice and for carers who regret not being present at the time of death.

“*I remember the day at the hospice when they decided they were going to stop visiting completely and they said at midnight everybody needs to have gone. I never thought in 2020 we would have people dying alone, it was the most shocking part of my career” (Staff 22)*.

“*I could have just stayed there [at the hospice], my biggest regret is they wouldn't let me stay with her. I didn't get it, not just for me there must have been other people going through the same thing” (Carer 12)*.

Many felt the decisions were not theirs to make and have struggled to come to terms with how many patients died alone. One carer was forced to make a decision between being with his dying partner at the hospice or their young child at home because only one person was allowed to go in.

“*At the hospice [son] wasn't allowed to go in, only one of us. So I stayed with him, he was going through it too, he needed me” (Carer 13)*.

#### Not having others around

For people dying at home, carers feared bringing the virus into the home and spreading it to the dying person.

“*You were only allowed so many people in the house, we were scared at the time” (Carer 4)*.

Many were also fearful of prosecution when visitors were not allowed.

“*My children couldn't come and see him because of the law” (Carer 9)*.

Unable to have friends and family visit and be present had consequential problems in getting support for both the dying person and the carer,

“*He had only arm's length contact with his friends, two great friends that would ring all the time. But there were days when he was just too poorly it was too much for him. I don't think he had emotional support” (Carer 14)*.

“*You couldn't say, just sit with your Dad whilst I go out for a bit, like we did when mum died, because the kids couldn't come over [to the house]” (Carer 6)*.

Similarly, people could not go out and see other people and gain any respite from the intensity of home care.

“*I might have gone to my friends and spoke to them but we couldn't. I might have gone to my mates and moaned and come back but we weren't able to” (Carer 3)*.

Not having others around extended to disconnection in service-led support. Many carers reflected on the difficulties of contacting GPs and social services, making their ability to “care” so much more difficult, which for some in turn affected their decisions about their place of death.

“*GP refused calls to the house. GP was a joke. We had to keep phoning, it wasn't great” (Carer 4)*.

“*Mum asked me to take her home [from the hospice], I said I would try my best but I was terrified I wouldn't get any help because I had spent so long trying to get help for her and I couldn't get any, and I was terrified it would just be me and her at home, her in pain and I wouldn't be able to get any help (crying). I had to say look how difficult it has been so far, the doctors won't come out, the GP won't come out, we would have had to have some other care in place but it was the thought it would get to the end and I would be there on my own and not know what to do” (Carer 11)*.

#### Robbed of memory-making

The restrictions on how people socialize affected how people could meet up with family or friends, travel, and engage in social and leisure activities. Carers felt they had limited options for making memories as they were not able to do the things they used to or organize the special events they might otherwise have held.

“*It was all shut down so you couldn't even do things to make memories...it robs you of memory making” (Carer 3)*.

The missed opportunities for attending funerals (due to limits on numbers) and lack of celebrations of the deceased's life are likely to have future psychological implications for those who have been bereaved.

“*His cremation was held in [region] and because of the travel restrictions we couldn't go and his ashes were delivered back to us by a chap in a van… With my mum's funeral we had everything, but for my dad we had nothing, that time to cry and support each other, have a laugh at the end with my father there's nothing” (Carer 3)*

As carers reflected on the things they would have expected to do and were not able to such as funerals, social gatherings and offering support to each other, many became upset. Carers recognized they had not fully dealt with the emotional distress of loss or that coming to terms with the death took longer due to the circumstances of the death during a pandemic.

“*I question myself still to this day, I feel guilty. I don't know why but I do everyday, I have regrets. I couldn't see her at the end, be with my mum by her side. I would have liked to have been by her side so I regret and feel guilty about that” (Carer 4)*.

“*I spent a lot of time writing to people which helped me get some closure. Telling people and having the response was helpful and that helped toward closure but that could have come much sooner I think but thanks to covid it took a long time” (Carer 8)*.

In the absence of funerals or reduced numbers at them, people creatively used technology in an attempt to memorialize the deceased but it was not what they would have planned if restrictions had not been in place.

“*We couldn't have a funeral so we did a playlist and a ‘raise a glass' where people could play the music and remember him. My brother went on skype to sit with my mum for the funeral so he could help her, she is older and forgetful” (Carer 10)*.

“*A disenfranchisement of bereavement. You've not been allowed the normal routines and rituals. They've missed out on that important part of somebody's death and that ritual around it” (Staff 5)*.

The quotes show how the missed opportunities of being with people, having others around, and restrictions on memory-making have caused distress and may have likely impacted coming to terms with loss in the usual ways we would expect as part of bereavement.

### Lone carer

#### High intensity

As preferences for place of care changed to the home, many family members and close persons became carers overnight, resulting in them becoming lone carer. Many had no previous experience in caring roles and had not expected to be in the position of being a carer when the illness was first diagnosed.

“*I had no clue about caring for somebody. I see myself as a caring person but practically wise in terms of actually trying to nurse somebody I've never done that” (Carer 11)*.

It was a time of high intensity, and many carers felt unprepared and ill-equipped for the role.

“*I phoned up [hospice] and said we need help, I've never ever been in this situation and never want to be in it again. We were so tired. I said we need help, we don't know what to do” (Carer 1)*.

This was an intense time for carers who took on the responsibility of managing the day-to-day changes associated with end-of-life care at home.

“*There was no escape from it, we couldn't go anywhere…I think it was probably much more intense [at home] than it would have been at the hospice” (Carer 3)*.

“*By this time I couldn't lift him or turn him” (Carer 14)*.

One carer reflected on her mother having a hospital bed arrive, and what she thought was a waterproof mattress cover turned out to be a sliding sheet for transferring a patient, but she did not know; no one had explained it, and she had not seen one before.

“*It turned out it was one of those rubber sheets they use to maneuver patients around, but I had no clue what to do with it how to use it, it was no use to me. I couldn't move her around on my own with no training or help. I hadn't got a clue” (Carer 11)*.

One of the hospice community services sent out training videos and leaflets about manual handling to carers which were crucial in helping them manage the day-to-day aspects of care.

“*I got a lot of information and an information pack from (the hospice), which was utterly invaluable at the end of the day… I wouldn't have known how to do a lot of things, so I can access things like little videos about how to lift. You know how to move people, so I was able to access all of that support” (Carer 6)*.

The intensity increased for carers when employers were not supportive, or did not understand the needs of carers, or bereaved.

“*My employer was very unsupportive. I asked to work from home to be able to be there for dad and they said no, until it was enforced by the government” (Carer 3)*.

“*I approached my employer about the possibility about being on furlough so I could spend time with my dad and my boss couldn't help me… in the end I just walked away. I should have found out my rights before, found out what my rights were. My manager didn't know” (Carer 5)*.

#### Reduced home support

Many staff took on additional roles, acting as a liaison between the dying person and healthcare professional, doing assessments over the phone, or taking on responsibility for managing strong medications remotely. As community support and social care became overwhelmed, care within the home became fragmented, with a reliance on agency staff and different care staff coming in daily, affecting continuity of care.

“*One young woman looking after her mother who was quite young as well, they just seemed so isolated. There weren't any family members because they were trying to do the right thing and keep away. So this girl was very dependent on services coming in to help her” (Staff 5)*.

“*It was a constant revolver of different people coming in and out, we had no idea who these people were” (Carer 13)*.

Contacting numerous agencies to get help, advice, and support seemed more problematic during the pandemic period. Many carers reflected on the difficulties of getting to speak to somebody, and professionals were reluctant to attend the home.

“*I remember spending lots of time on the phone to various people, hospice services, social services. [Hospice] advised me to contact social worker, but we didn't get anything back from social services until about 4-6 weeks after she died, it was too late, she had died” (Carer 11)*.

“*The difficulties with the GP surgery just made it unnecessarily hard. I can't complain about the district nurses or hospice nurses, they were very good… it could have been so much better” (Carer 14)*.

As face-to-face services stopped and social distancing and shielding were imposed, carers had reduced informal and formal support, people were dying at home, and many felt abandoned and isolated.

“*Emotionally I needed support and I didn't have any” (Carer 5)*.

“*Me being stuck at home all the time wasn't the best, losing that social interaction when we launched into another lockdown. We did form a bubble but it's not ideal” (Carer 13)*.

Staff expressed the concerns they had about carers taking on additional pressures at home

“*It adds isolation and disconnection” (Staff 1)*.

As Carer 11 described earlier, the fear of being left alone without support from her dying mother forced her to make the decision to remain an inpatient in the hospice. Staff acknowledged the potential impact forced decisions may have on bereavement processes and grief and the longer-term implications for bereaved carers.

“*I would imagine people for all sorts of reasons might have complicated grief, I mean the impression of feeling abandoned which a lot of people spoke about, the fact that lots of routine services were no longer there, the types of deaths, the way it happened, the way to media portrayed it, the fear. All of that, I don't think it's just going to go away” (Staff 4)*.

#### Increased quality time

For carers who felt supported, the forced privacy, ability to work from home, and lack of opportunities to do anything else resulted in quality time for the carer and the dying person. For some, the lockdown protected the dying person from the realities of having to say goodbye or doing things for the last time and instead gave carers and the dying person valuable quality time to be together.

“*He was private about these things so actually the pandemic helped him. Although it was intense, we were able to have that time together which was valuable” (Carer 14)*.

“*For some folk required to isolate during the pandemic it removed the pressure of having to socialize and having to go out into public, so that was helpful for some people” (Staff 1)*.

Carers felt very strongly that hospice services enabled quality time to happen, particularly the night service support which enabled the carer to get some much-needed sleep; the coordination of care support and the information packs given out about manual handling; and care at home, which provided much-needed practical advice where no other support was on offer.

“*Hospice nurse became the liaison for support. They were fantastic” (Carer 1)*.

“*When the hospice people came, they would spend more time [with us]… The hospice care felt more dedicated, staff didn't seem as rushed, I don't want to say more dedicated but they have a greater understanding of what is going on because it is specialized” (Carer 13)*.

At home, carers put their “own systems in place” to protect the dying person and try to improve their quality of life and support.

“*We had our own systems in place for deliveries, cleaning down everything. Our vicar would stand in the garden and talk to us” (Carer 12)*.

As the quotes have shown, the lone carer role was intense during the pandemic, with reduced support. Whilst some appreciated the quality time, many felt the increased burden of isolation and exhaustion, which again may likely impact wellbeing. Several interviews revealed that many carers had not come to terms with their grief, and it was only during the interviews that they took the time and space to reflect. For many, they were still in crisis mode of getting ‘through' and had not reached any level of acceptance of loss.

### Process vs. patient-centered care

#### Changing rules and regulations

Staff working in the hospice and community reflected on the rules and regulations put in place as a means to protect the workforce and patients. There was a focus on risk assessments to keep patients and staff safe, but rules changed rapidly—especially during the first lockdown—which required flexible working for staff and understanding for carers and patients.

“*All the changes that are going on all the time and having to work out new procedures and implement changes on the hoof! Having to do it very quickly, it's really difficult” (Staff 1)*.

“*Changes were coming at us everyday” (Staff 20)*.

As rules changed and restrictions were brought into the hospice, several staff reported the difficulties of having to ask relatives to leave, as well as the emotions they felt when they had followed the ‘rules' to not allow relatives to visit, but then other staff had let relatives come in.

“*We succumbed and more people ended up in the room (with the dying person at the hospice) but then the nurses were annoyed because two weeks prior, there was a young lady and four daughters and because they didn't put up a fight those four sisters weren't together with their mum before she died” (Staff 6)*.

Many staff felt they responded and reacted well to the changing rules and procedures during a crisis.

“*The responsiveness, the flexibility, the team working, the team, coming together phenomenal” (Staff 3)*.

However, some staff raised concerns that, despite their hard work, the procedures and restrictions challenged the concept of patient-centered care, and both inpatient staff and community staff were concerned about the impact on the experience of care for the patient and the carers.

Concerns about how the rules around wearing PPE and social distancing were impacting the delivery of palliative care were evident amongst the staff. Whilst staff understood the rationale, they expressed concerns about how the wearing of masks and visors made it difficult to communicate and identify the subtle cues associated with dying.

“*We're all wearing masks and gowns. I'm sure it's a physical barrier and how that affects communication and how we connect with people, how we read people's body language and expressions. It's more challenging hidden behind a mask” (Staff 1)*.

Staff were also worried about the impact of visitors wearing PPE in hospices.

“*Now when their wife and daughter are sitting with them, they have to wear a mask and a pinny. They've been married for years and at the end have to wear a mask just because he's here [hospice]. I think that's so sad” (Staff 21)*.

“*I wonder how many patients and relatives have struggled to hear what I was saying or have not been able to read my body language. From a staff point of view, we are all now so comfortable wearing PPE and masks all the time, I don't think about it when I'm talking to patients. I forget now I'm behind a barrier” (Staff 6)*.

#### Essential care

Care both within the hospice and in the community became process-led. The physical care was prioritized with “non-essential” care and services being stopped, such as massage therapies, reiki, and support groups, all highly rated by staff and carers. Face-to-face contact was minimized at the hospice inpatient unit and, in many cases (in England), stopped altogether in the home. Some staff felt the holism of palliative care was lost, and carers expressed they felt “isolated” and “neglected”, acknowledging a lack of mental health support with a focus on physical care.

“*We did the physical care we had to do. We could look through blinds, we could give medications but all throughout my career we've been taught holistic care. That the psychological and spiritual is every bit as important as the physical. But actually it felt that it was the physical that counted and everything else had to go by the wayside” (Staff 19)*.

In the hospice, carers felt frustrated at not being able to stay and worried people would die alone if contact was minimized,

“*No, nobody would pop in and see how she was. I said to her does anyone come in and see you at night, and she said no I don't see anyone. I complained about it and one nurse did take that on board but I was disappointed with that, I expected more than that” (Carer 12)*.

Contact within the home also felt minimized with a focus on the physical aspects of care

“*It was very much someone was coming in (to the home) to do a job, there was a distance there, we're coming in all PPE'd up we've come to do a test to ensure that medical she is okay. There was a lack of the mental support, not even mental health just general support, no emotional support someone sitting with you, having a chat and something would come out of it” (Carer 13)*.

For many staff, it seemed that care became system-led, which is not in line with the holistic practice of palliative care. Despite attempts to live with COVID-19, many systems of support important to people at the end-of-life and their carers have not been re-established.

“*Day therapy unit effectively shut down, nobody able to come in. Something I have missed hugely…We haven't had a fully functioning multi-disciplinary team for a while… We've reduced back to a medical and nursing model but palliative care is so much more than… it's not the full service that we want to provide and that people need” (Staff 5*).

“*It's socialization, it's using therapies like reiki, aromatherapy, I really miss that and the groups that were there. These things make people feel a bit more human. They take people out of their illness and give them a bit of normality” (Staff 15)*.

The lack of integration between health and social care seemed to exacerbate during the pandemic and does not seem to have resumed. Carers expressed their frustrations with getting prescriptions, contacting GPs, arranging appointments, and getting equipment or support in the home.

“*I still have a massive sense of frustration. I feel enormous frustration where this is the norm, and it's a really bad norm, many people, especially very elderly people can't cope with doing things online, they can't cope with waiting for 40 min on the telephone, if you're ill, it's a long time. I still have a massive sense of frustration” (Carer 14)*.

The impact of COVID-19 is evident in hospice care: missed opportunities, the overwhelming role of the lone carer, and the changing nature of palliative care as the process took priority. Even though some restrictions had been lifted at the time of the interview, practice as hospice staff knew it before has not resumed, taking a toll on the workforce and directly changing the delivery of palliative care.

“*Some services are still not running, face-to-face (particularly in the community) is not the first line of support, it is phone, then video, then face-to-face” (Staff 10)*.

Staff were concerned about the overwhelming demand, with some staff reducing hours or leaving hospice roles.

“*Support has been awful for doctors in training roles, never known so many junior doctors to have to go off on sick leave, it's really quite worrying” (Staff 9)*.

“*I'm exhausted, but then we all are” (Staff 10)*.

The longer-term implications for carers coming to terms with loss are evident through our findings. Whilst carers seemed to understand the restrictions in place due to the pandemic, many felt they had lost quality time and that it had taken them longer to come to terms with a close death due to the restrictions of the pandemic.

“*Covid has destroyed a lot of stuff... It's time we have lost now. For that last Christmas, we had to say no we can't do anything for Christmas because of risk of infections. Covid has been dramatically destroying, it's horrible” (Carer 4)*.

“*I would be surprised if anybody who lost anybody during the pandemic didn't have an issue with it… For 12 months I have been seeing a counselor. I've not slept properly at all, it's only just recently I've got any real sense of sleep... I'm in the second year, I've been through the firsts, the anniversaries, birthdays, and I've suddenly realized that I've made no memories with her last year, there's just emptiness” (Carer 14)*.

Carers have not had the ability to grieve in the traditional ways through social connections and networks. At the time of the interviews, many had not returned to living their lives the way they did pre-pandemic, with some remaining isolated. As a result, not all had the opportunity to experience the “firsts” that help in recovery, such as the first anniversary of the death or holidays and special events without their loved one present in social situations. It may well be that adaptation to life without the deceased has taken longer due to the lack of social opportunities. Consequently, future losses may manifest in different ways and likely cause momentous responses to grief.

The findings were shared with key stakeholders and policymakers in two virtual roundtable events (England and Scotland), and potential policy and practice implications were identified (Table 3).

**Table 3:** Policy roundtable discussions.

**(1) Technology has played an important role in providing a platform to stay connected, and for many, it has enabled services to continue remotely. Carers and staff acknowledge technology has a place in care, but should not replace human connection or the value of the therapeutic setting. Stakeholders at the roundtables raised the lessons learned about supporting people with technology in relation to access to devices and guidance for using them, and the values of hybrid systems, but acknowledged that some elements, such as communication about terminal diagnosis and final goodbyes with a person dying of a terminal illness, need face-to-face interaction**.
**(2) Sacrifices were made by carers and staff during a very challenging time**. For many carers, at the time of the interview, they were still yet to experience their important “firsts” associated with bereavement—the first birthday of the deceased, the first Christmas without the close person—because only recently had social connections been fully resumed. For many, the “lockdown” stopped exposure to the realities of social loss, and this may have implications for complex and prolonged grief, which may manifest in other ways. Stakeholders acknowledged the need for wellbeing and support for carers, including financial benefits and better support from employers and education settings for carers and the bereaved.
**(3) Health and Social Care staff have experienced significant challenges working in an unfamiliar pandemic context, in different roles, and working in different ways**. Hospice staff have tried to deliver quality palliative care despite pandemic restrictions, regulations, and fear. Stakeholders discussed the importance of staff recruitment and retention and ways in which the hospice workforce can be supported physically and emotionally, acknowledging more needs to be done to support recruitment and retention in hospice care.
(4) Our findings suggest that **carers have missed out on important life moments, that their experience of caring was a lonely one and that care became system-led and not in line with palliative care's ‘gold-standard' approach**. Stakeholders acknowledged that as a result, choice and quality throughout a person's terminal illness and end-of-life may have suffered. There is an opportunity to facilitate open conversations about dying, death, and bereavement, as whilst conversations significantly increased during the pandemic, there have been significant challenges in how these conversations took place. Stakeholders acknowledged that public attitudes and public opinions of health and social care needed to be rebuilt and that this will require a whole-system, integrated approach.

Findings are now discussed in relation to the wider literature and identify the implications for hospice care, workforce, and carer support following the roundtable discussions.

## Discussion and recommendations for policy, practice, and public health

This qualitative interview study aimed to explore the decision-making process about the place of end-of-life care during the COVID-19 pandemic and the impact on the experience of end-of-life care from the perspectives of bereaved carers and hospice staff. Our findings demonstrate a clear change in preferences, with decisions to provide care at home being heavily influenced by the restrictions on visiting people in hospices. As a result, family members took on the role of primary carer, and for some, this was unexpected and a role they were not prepared for. Through interviews with bereaved carers and hospice staff, the research has demonstrated that end-of-life care during the COVID-19 pandemic was challenging for several reasons. Hospice staff tried to retain the quality of palliative care through creative and alternative ways of working, but the restrictions imposed both within the home and the hospice inpatient unit made holistic, patient-centered care impossible. This is the first article to report in detail the rich experiences of the paid and unpaid hospice workforce providing end-of-life care during an unprecedented time of change and transition in palliative care. It offers valuable insights into how people experienced care and the impact it has had on life post-pandemic, with potential longer-term implications for the hospice workforce and bereaved communities.

The first theme of “missed opportunities” describes the increased emotional distress and responsibilities taken on by carers when they were not able to visit the hospice and people were not able to visit those dying at home. Social restrictions meant that carers missed out on opportunities to make memories or memorialize the death of someone close. In the second theme, the experience of the “lone carer” highlights the level of commitment and high emotional and physical intensity of being a carer in the home when healthcare services were limited to remote or digital contact, reduced visits and, in some cases, withdrawn altogether, At the same time, for some, we found that taking on caring responsibilities and isolation resulted in quality time as death neared that people were otherwise unlikely to have experienced. The third theme explored how staff struggled to balance clinical and physical processes of care with the more holistic patient-centered approach of palliative care. This described the changing nature of end-of-life care as a result of the rules and regulations in place and how adherence to regulations such as visiting and wearing PPE was prioritized over the individual care needs of terminally ill people and their families, resulting in dissatisfaction for both carers and staff.

The better end-of-life research report (4) suggests that the changes in where people died and who was receiving service-led palliative care were not a direct consequence of the COVID-19 infection, but resulted instead from indirect consequences of the pandemic, such as changes in the way people accessed services and disruptions to the health and care system. As a result, they propose a need to invest in primary care, community, and palliative care services to ensure high-quality and equitable care at the end-of-life to meet the growing demands. However, our study shows that during the pandemic crisis, end-of-life care was forced to be delivered differently and in alternative ways, with a focus on physical care and not the holistic approach associated with high-quality palliative care.

The impact of COIVD-19 on Hospices (ICOH) study (19) is the only other study to have used in-depth interviews with carers in a hospice context. They similarly found that despite many of the best efforts of frontline healthcare professionals, hospice care was compromised and fell below the “gold standard” expected (19). The study also included patients and offers further support for our findings that all those who came in contact with the visiting restrictions (patients, cares, and managers) experienced emotional distress. This study also recognizes that bereavement during the pandemic could be challenging for carers and that many hospice staff were left emotionally and physically exhausted, raising the question of long-term burnout and recommending the provision of mental health support for staff. Both studies demonstrate that significant individual costs are experienced by family members and close persons who were denied the opportunity to be with someone at the end-of-life. Both the carers and hospice staff who participated in our study shared the deep emotional impact of what they have experienced, and we, as a society, need to learn from the COVID-19 pandemic to ensure that in the event of a future health crisis, people are not denied such opportunities.

Other studies have explored staff experiences and found similar results to our research. In a qualitative study of staff working in palliative care, Bradshaw et al. (20) identified that despite their experience of dealing with death and dying, the mental health and wellbeing of palliative care staff were affected by the pandemic. Bradshaw suggests that organizational, structural, and policy changes are urgently required to mitigate and manage longer-term impacts for staff. Hanna et al. (21) conducted a qualitative study with healthcare professionals working during the pandemic across a range of settings and found both emotional and practical challenges to providing end-of-life care during the pandemic, including increases in patient numbers, reduced staffing levels, and relying on virtual platforms for sensitive, emotive conversations with relatives. Similarly, in Italy, Franchini et al. (22) found that staff providing home palliative care services faced both patient-related and practice-related challenges, which required different communication methods and patient and family education, but staff felt a sense of satisfaction with their role during the first wave of the pandemic. In a hospital setting in the USA, Vesel et al. (23) found that palliative care served as a bridge between providers, patients, and families and reinforced positive perceptions of palliative care.

Situating our findings within the wider literature and policy reports, there are two overarching themes for further consideration: (1) future of caring and (2) a public health concern.

### Future of caring

Every year, 4.3 million people become unpaid carers, 12,000 people a day (5). Our study has shown that throughout the pandemic in both England and Scotland, people took on new carer roles that they were not prepared for, which had a significant impact on their physical, emotional, and financial wellbeing. Carers are crucial in helping terminally ill people get the day-to-day support they need for a good quality of life. Identification of carers remains the primary barrier to carers accessing the support they need and are eligible for in their caring role, whether physical, emotional, digital, or financial. We, therefore, recommend ways to identify carers that are backed by educational support, such as carer toolkits and public health campaigns (see Table 4).

**Table 4:** Recommendations and implications for public health, practice, and policymakers.

**Issue**	**Need**	**Recommendations**	**Responsibility**
Identification of Carers	To improve carer identification and pathways, backed by educational and support resources for carers, health and social care staff, employers, and education settings.	1. Creation of a toolkit for carers new to a caring role, coproduced with carers, and condition-specific training and support for carers looking after a terminally ill person with complex, condition-specific needs including dementia, given the number of deaths from dementia as the primary cause of death will have increased by 185% by 2040. 2. Sustainably funded, national public awareness campaigns must be undertaken by policymakers highlighting the diversity, invisibility, and prevalence of carers with a focus on carer self-identification	National and Local Governments, Integrated Joint Boards (Scotland), Integrated Care Boards (England)
Assessment of carers support needs	Technology has played a significant role for carers and hospice staff during the pandemic, but there have been significant challenges in accessing technological devices and guidance for usage.	1. Carers assessments must include digital and financial needs assessments, and in recognizing the progressive nature of terminal illness, all carers must be offered a Carers Assessment at least annually, with prompt follow-up after initial invitation. 2. CSNAT - Carer Assessment Support Needs Tool – should be used to further support the identification of carer needs once registered in statutory systems, and signposting relevant CSNAT palliative care resources.	Local Authorities. Integrated Joint Boards (Scotland), Integrated Care Boards (England)
Carer Support		1. All health and social care professionals, employers, education settings, and community link workers should complete certified ‘Carer Aware' training, including how to provide information and support to carers and signpost information, with the option for flexible working or studying patterns for those with caring roles. 2. Fast track Carers Support Plan or Young Carers Statement if caring for someone who is terminally ill, and proactively signpost to support effective, clear communication with carers. 3. Establish a UK Carers Research Network, to better understand decision-making and long-term implications of COVID-19 on changes in end-of-life care preferences. 4. Eligibility criteria for carer breaks should also be removed, and carers must automatically be offered support for breaks from caring.	Health and social care workforce, employers, and education providers. National Governments and Academic Institutions. Local Authorities
Essential Carer role		The ‘Essential Caregiver' status must be maintained beyond the pandemic, with coproduced, standardized rights for carers in all health and care settings.	National Governments
Prioritize carer statutory services		1. Prioritizing statutory services to meet the physical, emotional, and financial needs of carers, starting with a return to pre-pandemic levels 2. Prioritizing the reopening of Carers Respite Centers alongside NHS services in future health crises	National and Local Governments
Financial support for carers		1.The rate of Scottish Carers Assistance must be extended to England, Wales, and Northern Ireland and updated annually in line with inflation 2. Scottish Carers Assistance and Carers Allowance must be extended to 6 months after a bereavement	National Governments
Isolation and loneliness of carers Fear, which carers continue to experience, and has resulted in a lack of reintegration into society whilst still living with COVID-19		1. Clear and accurate public health messaging should be embedded in post-pandemic and future health crisis planning. This should include clearer, and more timely Government communication around changes in any future restrictions, and more agile risk management to reflect the support needs of carers of terminally ill people where physical communication is a core aspect of their care, including dementia and Motor Neurone Disease 2.Widening Anne's Law in Scotland to include a permanent, dedicated visitor (which could be a carer) for terminally ill people in all care settings 3. An ‘inclusion health' approach to physical, emotional, and financial support carers need 4. A human rights approach to bereavement to tackle the isolation and loneliness of carers	National and Local Governments

Support for carers is often overlooked (5); our study emphasizes that this was exacerbated due to the restrictive measures associated with the pandemic, and therefore carers need to be more greatly recognized within society. Whilst numerous studies have explored the impact of the COVID-19 pandemic on carers (16) and healthcare workforce (5, 11), our study focuses on the end-of-life. Our findings collaborate the study of Mitchell et al. (24), who conducted a UK-wide survey of community services from people at the end-of-life during the first wave of the COVID-19 pandemic. Similarly, Mitchell et al. (24) identified that services adapted rapidly to meet the increased number of patients in need and to address the complexity of people's needs at the end-of-life. With more people wanting to remain at home due to fear of the virus and not being able to visit, as shown in our study, the needs in the community increased. Mitchell et al. (24) also showed that the community took on greater responsibility for most areas of palliative care clinical practice, including GPs; however, in our study, carers appeared frustrated at being unable to access their GP and community services. Our study includes in-depth data that shows the distress to carers due to breakdowns in communication between services and the intensity of burden and isolation during the pandemic. Carer breakdown and burnout are the most likely factors in a person with a terminal illness being admitted to a hospital, hospice, or care home, and having a live-in carer is one of the most important factors in whether someone is able to die at home or not (25). Assessing carer needs must extend to digital, financial, and emotional needs, and we recommend that tools such as CSNAT may be helpful in assessing carer needs (see Table 4), and assessments should be conducted regularly to capture adaptation and change as the end-of-life nears (26). The expectations of the carer have increased significantly during the pandemic for terminally ill patients and health and social care professionals. More agile risk management must be embedded to reflect the support needs of carers of terminally ill people with conditions where physical communication is a core aspect of their care, e.g., dementia and motor neurone disease.

Some carers in our study highlighted the difficulties of managing work and caring roles, with some people leaving work to car full time. Where employers had been supportive in allowing time off and where people had been able to take furlough or work from home flexibly, they were able to manage paid work with their carer role. We, therefore, have several recommendations for employers and education providers to be ‘carer aware'; to fast-track carers support places; and to establish a carers research network to better understand the decision-making and long-term implications of the COVID-19 pandemic on changes in end-of-life care preferences.

### A public health issue

Our findings show that the pandemic exacerbated uncertainty and anxiety, communication challenges in healthcare settings, and isolation and loneliness in carers caring for people at the end-of-life, supported by literature for carers with chronic ill health (16). Inclusion health is an umbrella term used to describe vulnerable or socially excluded groups who experience multiple overlapping factors impacting poor health, including poverty, complex trauma, and multiple disadvantages (27). Poor access to health and care services and negative experiences can also be commonplace for inclusion in health groups due to multiple barriers, often related to the way healthcare services are delivered. People belonging to inclusion health groups frequently suffer from multiple health issues, including mental and physical ill health and substance dependence issues. This leads to extremely poor health outcomes, often much worse than the general population, a lower average age of death, and it contributes considerably to increasing health inequalities. People in inclusion health groups experience stigma and discrimination and are not consistently accounted for in electronic records, including health databases. Inclusion health includes any population that is socially excluded. Whilst this typically includes population groups that experience acute social exclusion, including people experiencing homelessness and people in contact with the justice system, amongst others, there is a case that carers should be reflected in such population groups. An inclusion health approach to the physical, emotional, and financial support carers needs to facilitate their reintegration into communities and society should include clear, concise public health messaging that is easily accessible.

We have several recommendations to promote inclusivity for end-of-life care across the UK based on our findings. First, across the UK, the “Essential Caregiver” status must be maintained beyond the pandemic, with a coproduced, standardized set of rights for carers in all health and care settings, including communication, physical, emotional, and financial support needs, information, and involvement in decision-making from all health and social care providers. In Scotland, this could include widening Anne's Law to include a permanent, designated visitor (which could be their carer) for terminally ill people in all care settings, not just care homes, to ensure no one is prevented from having the opportunity to be with a dying person. Carers should be able to access respite care when needed, as well as information and peer support through carers' centers, and others, to enable them to continue to provide care to the person they are looking after and maintain their own wellbeing. Second, in the short and medium term, statutory services in the UK should be prioritized to meet the needs of carers, starting with a return to pre-pandemic levels. For future health crises, reopening carers respite centers alongside NHS services must be implemented simultaneously.

In Scotland, the development of a National Care Service (NCS) is scheduled to redefine how social care is delivered to those who need it. There are opportunities and challenges for palliative care within the NCS, but carers must be involved in the meaningful co-production of social care services and systematic processes that meets their needs and includes participation costs. This includes participation costs. In England, this includes establishing a Carer's Charter, and in Scotland, this includes refreshing the 2018 Carer's Charter to support and implement a new set of standards that improve carer's rights and “caring conditions”, and including carers on IJBs and subsequently new NCS Care Boards with voting rights. Third, increased financial support for carers, aligned with annual inflation, should be incorporated into policymakers' national and local budgets. The role of a carer has a huge impact on the life of the person they are caring for. Costs for carers are usually categorized into three main areas and have risen sharply during the pandemic (28):

1. Work-related (e.g., changes in employment such as going part-time or giving up work entirely).
2. Carer time costs (related to time investment required by carers).
3. “Out of pocket costs” (direct outgoings including transport, food, and medicines).

Across the UK, the value of unpaid care was estimated at £530 million per day and £193 billion per year during the pandemic (29). The cost of living crisis and the current economic recession are placing a disproportionately heavy burden on carers to continue absorbing significant caring costs. Whilst there has been some financial support from national policymakers, it is essential that policymakers recognize that financial insecurity, economic uncertainty, stress, and increased burden are forcing carers onto or below the poverty threshold.

Finally, national and local policymakers should adopt a human rights-based approach to bereavement to tackle isolation and loneliness, including the alignment of national and local strategies relevant to carers. When a caring role ends as a result of the death of the person being cared for, sadness and grief can make dealing with everyday life a challenge. The pandemic changed the dying; people were not able to visit or be with the dying person, funerals were limited or not able to go ahead, and mourners could not grieve together in person. As a consequence, the way people have grieved has changed. Whilst we understand that people experience grief and bereavement at different stages, our study highlights that bereaved carers were not able to grieve, come to terms with their loss, and memorialize the deceased as they would have expected if the pandemic had not occurred (30). Grief has likely been prolonged and, in some cases, more complicated due to restricted social connections, a lack of opportunity to memorialize, and for some, the sheer distress of how people died without adequate support and human connection. Whilst further longitudinal research is needed to confirm, the impact on families is likely to be damaging, with a significant increase in levels of grief and bereavement as well as more complicated grief. We, therefore, recommend that further physical, emotional, and financial support be extended to carers during bereavement. Extending the eligibility of carer's allowance to six months after a caring role ends is recommended, and this should be replicated in the upcoming Scottish Carers Assistance and in England. We also recommend that the UK “Tell us Once” service be extended to Scotland, where bereaved people can report a death to relevant bodies in one instance, rather than recounting the experience multiple times and reliving trauma.

### Limitations

Although qualitative research does not seek generalizability, our sample is limited in that it was drawn from those accessing specialist palliative cares. More research will be helpful in other settings to give a broader picture of the impact across settings. Our sample was also largely white and British, which is a limitation in terms of the diverse populations in England and Scotland. Enhancing the diversity of the participants from other ethnic and cultural groups will be helpful. We would suggest further research to study in-depth the experiences of people in diverse communities and people experiencing structural inequalities who may feel they are unable to access professional services such as hospice care. This may include people who are homeless, people who experience discrimination, or people who see unfair distribution of health and social care opportunities. It is important to provide evidence of a broad view of people in the whole of society if policy and practice change is to be sustainable and effective. Studies following the same research design but in different countries with different “lockdown” guidance and regulations would also be useful to compare the longer-term implications on health and wellbeing.

## Conclusion

The COVID-19 pandemic has undoubtedly had a significant change in place of care and decision-making at the end-of-life. The impact on health and social care services (and communities) is immense (i.e., wellbeing, employment, and mental health), and the full extent is likely not yet fully visible. It is likely that the social restrictions in place may mean that some have experienced delayed grief, but further research over a longer period of time will be required to explore further. The findings from this study have key recommendations for policy, practice, and public health to support carers in future. A public health approach to care, where health, social and community systems work together is essential. For staff, the exhaustion associated with working through a pandemic is evident. There were some excellent examples of care delivery, working collaboratively, and communicating well, but if the core concept of palliative care is not restored, our workforce will further fragment. Public opinion has changed, and managing societal expectations of health and social care will be challenging. Clear public health messaging and actions are necessary, along with sustainable educational resources and support for health and social care professionals, carers, and citizens.

## Data availability statement

The raw data supporting the conclusions of this article will be made available by the authors, without undue reservation.

## Ethics statement

The studies involving humans were approved by Science, Technology, Engineering, and Mathematics Ethical Review Committee, University of Birmingham. The studies were conducted in accordance with the local legislation and institutional requirements. The participants provided their written informed consent to participate in this study. Written informed consent was obtained from the individual(s) for the publication of any potentially identifiable images or data included in this article.

## Author contributions

CB, PG, JM, AF, RM, EW, and SS: conceptualization and writing. CB, PG, JM, and AF: methodology. CB and PG: formal analysis. All authors contributed to the article and approved the submitted version.

## Supplementary material

Data Sheet 1 Staff Interview Schedule.

Data Sheet 2 Carer Interview Schedule.

Data Sheet 3 Distress Protocol.
