# Supplementary material for: “Palliative care is so much more than that”: a qualitative study exploring experiences of hospice staff and bereaved carers during the COVID-19 pandemic
Source: Front Public Health. 2023 Oct 25;11:1139313. doi: 10.3389/fpubh.2023.1139313 (PMC10662348; doi:10.3389/fpubh.2023.1139313)
Supplement: Data Sheet 1 — Staff Interview Schedule. [file Data_Sheet_1.docx]

Interview Schedule – Staff

**Introduction**

Welcome and introductions

- Thank you for your time

Overview of the study

- Did you read the information sheet?
- Do you have any questions?

- This study aims to investigate how the pandemic has impacted on hospice services in terms of place of care preferences and the experiences of carers. Our research aims to explore how the pandemic may have changed your perceptions and/ or experiences of hospice and care services. So I have a few questions to ask you about your experience of being an carer. There are no right or wrong answers; we are interested in what you have got to say and your experiences. You can stop at anytime.

Screening check for exclusion criteria (Staff working in MC / Nursing services during pandemic)

Consent – virtually via survey monkey

OK to record / live transcript

**Demographics**

1. Staff Role (any redeployment / change to role during the pandemic?)
2. Experience
   - years qualified
   - time in current role
3. Gender classification
4. Age range group
5. Ethnicity

**Topic questions**

1. Can you describe how the covid 19 pandemic has impacted on hospice care services?
2. In your experience, how do you feel the pandemic has impacted on the care your provide to patients and families?
   1. Anything specific that has changed to your role?
   2. Or changes to the service?
3. How do you feel it may have affected how patients and their carers make decisions during this time?
   1. In particular around preferences for place of end of life care and place of death for those receiving hospice care?
   2. What about the impact of the visiting restrictions on decision-making and on experience?
   3. Do you have any specific examples?
4. What about the impact for people who are carers and those who have been bereaved during the pandemic?
   1. Do you have any examples?
5. How well do you think our patients and carers were supported during the pandemic given all the constraints?
   1. Has anything been learned?
   2. Any suggestions around how we would approach care delivery if in the same situation again?
6. In your opinion, what do you think Marie Curie needs to do next to improve the quality of care and services for:
   - Patients
   - Caregivers
   - Bereaved

**Debrief and summary**

Summarise key components of what has been discussed.

1. Do you agree?
2. Do you want to add anything further about how the pandemic has affected decision-making, preferences and experiences of hospice care for patients and carers?

Thank you for your time, this has been so very helpful to our research.

Explain how to claim honorarium.
