# Supplementary material for: “Palliative care is so much more than that”: a qualitative study exploring experiences of hospice staff and bereaved carers during the COVID-19 pandemic
Source: Front Public Health. 2023 Oct 25;11:1139313. doi: 10.3389/fpubh.2023.1139313 (PMC10662348; doi:10.3389/fpubh.2023.1139313)
Supplement: Data Sheet 2 — Carer Interview Schedule. [file Data_Sheet_2.docx]

Interview Schedule – Close Persons and bereaved carers

**Introduction**

Welcome and introductions

- Thank you for your time

Overview of the study

- Did you read the information sheet?
- Do you have any questions?

- This study aims to investigate how the pandemic has impacted on hospice services in terms of place of care preferences and the experiences of carers. Our research aims to explore how the pandemic may have changed your perceptions and/ or experiences of hospice and care services. So I have a few questions to ask you about your experience of being an informal carer. There are no right or wrong answers; we are interested in what you have got to say and your experiences. We can take a break at any point.

Screening check for exclusion criteria

- Adult, bereaved, close person who used hospice/ MC nursing services during the pandemic, been an informal carer between October 2020 – March 2021.

Consent

- Online via survey monkey

**Demographics / Context**

1. Gender classification
2. Age range group
3. Ethnicity
4. Role and relationship to patient
5. Services accessed and for how long
6. Context of patients diagnosis/cause of death
7. Length of time receiving hospice or community palliative care services

**Topic questions**

1. Can you start by telling me your experiences of marie curie services?
2. Do you feel the pandemic had any impact on your experience of caring for XX at home?
3. One of the things we wonder is how the pandemic may have impacted on peoples decisions about where they wished to be cared for,
   1. Did it affect your/ XX decision making in anyway in terms of place of care or
   2. Who visited the home? Impact of visiting restrictions?
   3. Readiness to access GP/Hospice/other services for advice support
   4. Access to out of hours support?
4. What about support for you?
   1. We are aware that support comes in many forms, e.g. formal through the NHS and hospice services and informal through friends and family, who supported you?
   2. Was this restricted / changed in anyway due to the pandemic?
5. Can you recall any examples where you felt the way in which we have had to live our lives during the pandemic may have impacted on your ability to care for your relative/ close person /name?
   1. If the pandemic had not occurred, do you think you may have had a different experience or done things differently in any way?

**ICECAP-CPM**
I’m going to put a slide up, each containing a question about the opportunities you had whilst caring for the person during the pandemic. These include questions about communication, privacy, practical and emotional support, preparing and coping and distress. I would like you to consider the question from your own experience. Each question has 5 possible answers from your ability to have or experience these things fully or all of the time to having them restricted or completely unable to. I will put a slide on the screen for each question and all the possible answers to make this easier for you. I am interested in your experiences. When you are considering each one, could I please ask you to verbalise you thoughts. This is called ‘thinking aloud’. It enables me to understand a little more about your response and gives me more insight into the challenges or support you experienced.

See slides:

1. Communication with those providing care services
2. Privacy and Space.
3. Practical Support.
4. Emotional Support. T
5. Preparing and Coping.
6. Emotional Distress to you, related to the condition of the person.

*Remind to ‘think aloud’

**Debrief and summary**

Summarise key components of what has been discussed.

1. Do you agree?
2. Do you want to add anything further about how the pandemic has affected decision-making, preferences and experiences of hospice care for patients and carers?

Check they are OK post interview. Follow distress protocol if required at any point during or after the interview.

Thank you for your time, this has been so very helpful to our research.

Thank you for your time, this has been so very helpful to our research.

Explain how to claim honorarium.

Explain how to claim honorarium.
