# Supplementary material for: “Palliative care is so much more than that”: a qualitative study exploring experiences of hospice staff and bereaved carers during the COVID-19 pandemic
Source: Front Public Health. 2023 Oct 25;11:1139313. doi: 10.3389/fpubh.2023.1139313 (PMC10662348; doi:10.3389/fpubh.2023.1139313)
Supplement: Data Sheet 3 — Distress Protocol. [file Data_Sheet_3.docx]

**Distress Protocol for staff participants**

**Identifying distress**

The participant indicates they are experiencing distress or exhibits behaviours suggestive that they are distressed (e.g. falters, stops speaking, persistent hesitation, tearful)

**Stage 1 response**

Check if participant wants to continue or if they would like a break.

Ok to continue – continue interview

Would like a break: The researcher stops the interview and pauses the recording. The interviewer lets the participant know they are no longer being recorded. Proceed to Review.

**Review**

Listen to the participant, provide space and time. Empathise.

After a few minutes, the interviewer asks the participant if they would like to carry on or stop.

Carry on: If the participant feels able to carry on, resume the interview.

Stop: If they don’t, discontinue the interview and move on to the stage 2 response.

**Stage 2 response**

Ask the participant if they think they might benefit from any support.

Yes:If they do require ongoing support, move on to the stage 3 response.

No: Move to Follow-up

**Stage 3 response**

Encourage participant to seek support from their usual contact point e.g. line manager or colleague. Let participants know they can also self-refer to get support from a Mental Health First Aider via the Marie Curie intranet. Move to Follow-Up

**Follow up**

If participant consents, follow up with a courtesy phone call within one week.

**Distress Protocol for bereaved carers**

**Identifying distress**

The participant indicates they are experiencing distress or exhibits behaviours suggestive that they are distressed. (e.g. falters, stops speaking, persistent hesitation, tearful)

**Stage 1 response**

Check if participant wants to continue or if they would like a break.

Ok to continue – continue interview

Would like a break: The researcher stops the interview and pauses the recording. The interviewer lets the participant know they are no longer being recorded. Proceed to Review.

**Review**

Listen to the participant, provide space and time. Empathise.

After a few minutes, the interviewer asks the participant if they would like to carry on or stop.

Carry on: If the participant feels able to carry on, resume the interview.

Stop: If they don’t, discontinue the interview and move on to the stage 2 response.

**Stage 2 response**

Ask the participant if they think they might benefit from any support.

No: Move to Follow-up

Yes: Offer to email a list of support contacts, internal and external to Marie Curie and move to follow-up. OR if the person is very distressed, move to Stage 3.

**Stage 3 response**

Ask if they would like a referral to the Listening Ear service. Move to Follow-up

**Follow up**

Ask if they would like a follow-up call in 7-14 days

Yes: follow up with a courtesy phone call within one week.

No: Thank the participant and close the interview.
